# Selection of dual-channel supply chain cooperation mode of older adults care service under the government subsidy strategy

**DOI:** 10.1371/journal.pone.0320741

**Published:** 2025-05-12

**Authors:** Tong Zhao, Qiaoming Hou

**Affiliations:** School of Management, Shenyang University of Technology, Shenyang, Liaoning, China; Landmark University, Nigeria

## Abstract

This study develops a dual-channel supply chain coordination model for services aimed at older adults, taking into account differentiated government subsidies. Utilizing Hotelling and Stackelberg game models, we systematically examine optimal strategies across three distinct scenarios: a non-cooperative mode, cooperation between online channels and logistics suppliers, and a tripartite collaboration involving both online and offline channels alongside logistics suppliers. The results demonstrate that the optimal pricing and service levels attained in cooperative scenarios exceed those observed in non-cooperative settings. Furthermore, within the framework of tripartite collaboration, the influence of enhanced service levels on the optimization of both service pricing and quality is particularly significant. It is noteworthy that government subsidies tend to exert a marginally greater incentive effect on offline service channels compared to online ones, thereby increasing the focus on addressing the emotional needs of older adults. Overall, this research represents a pioneering effort to compare these three service cooperation models, leveraging government subsidies as a catalyst. It not only enhances the advantages of differentiated dual-channel services but also promotes the efficient allocation of resources in elder care through the identification of suitable collaborative strategies.

## 1 Introduction

According to the 2023 National Ageing Development Bulletin, by the end of 2023, the proportion of China’s population aged 60 and above will reach 21.1% [1]. According to the National Bureau of Statistics, at the end of 2024, 22% of China’s population was over 60 years old [2]. This trend signifies a substantial expansion in the potential of the older adult market. The advancement of contemporary information technologies, including the Internet, big data, and artificial intelligence, has facilitated the swift emergence of the smart elder care industry, which is increasingly recognized as a significant catalyst for the growth of the silver economy market [3].

The positive view of aging believes that the current older adults have transitioned from focusing on quality of life to focusing on both quality of life and quality of living, and “needs-based” has gradually changed to “rights-based” [4]. This also means that meeting the increasingly diversified needs of older adults is not only in line with the trend of social development, but also the basic protection of the legitimate rights and interests of older adults. At present, China’s older adults population is in an inverted pyramid structure [5]. The application of IoMT and sensors, as well as other technologies, makes the existing intelligent older adults products and older adults services more suitable for the health care and medical care of older adults [6,7]. However, the deeper needs of older adults in addition to life care and medical care are often overlooked. Social-emotional choice theory points out that as life expectancy decreases, older adults pay more attention to emotional satisfaction than information acquisition, and their needs change from future-oriented to emotion-oriented [8]. Therefore, older adults care services need to be adapted to this preference to meet the multifaceted needs of older adults and protect their rights and interests.

The affirmative perspective on aging posits that contemporary older adults have shifted their focus from solely prioritizing quality of life to encompassing both quality of life and quality of living. This transition reflects a movement from a “needs-based” approach to a “rights-based” framework. Consequently, addressing the increasingly diverse needs of older adults aligns not only with the trajectory of social development but also serves as a fundamental safeguard for the legitimate rights and interests of this demographic. Currently, the population of older adults in China exhibits an inverted pyramid structure. The IoMT, sensors, and other technological advancements has enhanced the suitability of existing intelligent products and services for the healthcare and medical needs of older adults. Nevertheless, the deeper emotional and social needs of older adults, beyond mere life and medical care, are frequently overlooked. According to social-emotional choice theory, as life expectancy diminishes, older adults tend to prioritize emotional satisfaction over the acquisition of information, leading to a shift in their needs from future-oriented to emotion-oriented. Therefore, it is imperative that care services for older adults are adapted to accommodate these preferences in order to effectively address their multifaceted needs and safeguard their rights and interests.

In the process of developing the smart older adults care industry, providing applicable pension services for the older adults is an important task that the government needs to complete [9]. In 2015, the US government established the “Connected Care Pilot Program” aimed at providing financial subsidies for home medical services, thereby broadening access to these essential services. Concurrently, the UK introduced the “Smart Home Innovation Program,” which employs the loMT to support elderly individuals living independently, thereby improving their quality of life and mitigating the risk of accidents. Similarly, the Chinese government has allocated financial resources to various elderly care initiatives, including the “398 Smart Elderly Care Call Center” in Shanxi Province, the “Family E-Union” in Shandong Province, and the development of smart nursing homes in Shanghai. Collectively, these programs are funded through government subsidies, establishing a model of smart older adults care services that is characterized by government leadership and community involvement.

While the government has implemented targeted financial subsidies for elderly care initiatives, which partially address the care requirements of certain older adults, a persistent imbalance between supply and demand remains evident. The development of smart elderly care platforms and community-based institutional care continues to encounter issues related to resource inefficiency. Furthermore, the financial subsidies provided by the government at the level of the elderly care service supply chain are notably limited. In light of these challenges, this paper builds upon the framework proposed by Zhao and Hou [10] by incorporating the concept of user time perception into the coordination model of the smart elderly care supply chain. This study introduces government subsidies that are tailored to the characteristics of dual-channel differentiated services for the first time and conducts an analysis of optimal strategies across various cooperation models. This methodology not only facilitates government subsidies to encompass a range of initiatives, including the smart older adults care service platform and the development of older adults care communities, but also enhances the motivation of stakeholders involved in older adults care, promotes the integration of resources among all parties, and ultimately improves service efficiency.

The structure of this paper is as follows: Part II is a literature review. Part III introduces the background of the problem and defines the relevant parameters. Part IV constructs a dual-channel supply chain model of smart older adults care services under different cooperation models. Part V is analyzed by means of examples. Part VI provides management recommendations, and Part VII summarizes the findings.

## 2 Literature review

### 2.1 Older adults care service supply chain that considers the older adults needs

Contemporary research primarily focuses on the supply chain of secondary older adults care services, which encompasses both providers and integrators [11,12]. The central emphasis of this research is on the coordination effects and the quality of services within this supply chain. Zhao [13] developed a dual-channel medical care service model for older adults, which categorizes services according to the health status of the older adults and explores various pricing strategies. Additionally, Zhao [14]and Nguyen et al. [15] highlighted that the unpredictability of pension demand exacerbates operational risks for pension service integrators, potentially leading to information distortion. This disparity between supply and demand may jeopardize the sustainability of the older adults care supply chain. Glendinning [16] proposed that the incorporation of private enterprises could enhance the diversity of older adults care services, thereby addressing a broader spectrum of needs. Furthermore, Zhao et al. [10] introduced a dual-channel supply chain model for smart older adults care services for the first time, which prioritizes the learning and growth needs of older adults while also providing emotional companionship. This model aims to optimize resource allocation through an integration of online and offline services.

### 2.2 Introduce the supply chain of older adults care services in government departments

Numerous scholars have underscored the importance of government support in fostering the development of the smart older adults service supply chain, positing that effective oversight and policy initiatives are instrumental in advancing the pension industry. For instance, Mo et al. [17] employed blockchain technology to enhance information collaboration among government entities, pension service providers, older adults, and their families, thereby facilitating a more balanced exchange of information among all stakeholders. Li et al. [18] contended that the dynamism of the smart older adults care sector could be invigorated through government oversight and financial incentives, presenting a two-sided evolutionary game model to illustrate the interaction between pension enterprises and governmental bodies. Zhou et al. [19] developed a game-theoretic model examining the relationship between the government and private older adults care institutions, emphasizing the concepts of a “promising government” and an “effective market.” Their case analysis revealed that governmental support for the older adults care industry could be realized through policy and financial assistance, with an emphasis on enhancing the software and hardware capabilities of private care institutions as a manifestation of a “promising government.” Additionally, Shi et al. [20] proposed a tripartite game model involving platforms, older adults, and government entities to improve regulatory frameworks. Furthermore, research by Wei et al. [21] and Guo et al. [22] corroborated that government subsidies directed towards older adults care service integrators and suppliers can significantly enhance the quality of care services and augment the overall revenue within the older adults care service supply chain.

### 2.3 Basic principles and application design of game models

Hotelling [23] introduced an economic framework for understanding spatial competition, commonly referred to as the Hotelling model. This model posits that consumers are evenly distributed along the interval [0,1], allowing for a comprehensive analysis of pricing strategies and market equilibrium in competitive contexts. Chen [24] utilized the principles of the Hotelling model to develop a fixed demand function within the market, subsequently exploring issues related to supply chain coordination and optimization amidst horizontal price competition. Jiang et al. [25] applied the Hotelling model to investigate the effects of valuation disparities and channel preferences on two-stage competition for innovative products across three distinct pre-sale models, ultimately identifying the optimal supply chain strategy. Additionally, Zhang et al. [26] leveraged the Hotelling framework to assess the influence of offline transportation costs and online search costs on the service levels of dual-channel supply chains.

Beyond the Hotelling model, the Stackelberg game model is frequently employed by researchers to tackle issues related to strategy formulation, market equilibrium, pricing, and coordination optimization within supply chains. Liang et al. [27] developed a two-layer Stackelberg game model to analyze the effects of government subsidies on green supply chains in the context of meteorological disasters. Similarly, Ma et al. [28] utilized the Stackelberg game model to compare and evaluate coordinated optimization strategies within the older adult care service supply chain, focusing on revenue-sharing and cost-sharing contracts.

### 2.4 Research gaps and research contributions

According to the pertinent literature and the information presented in Table 1, the findings reveal several key points. Firstly, a significant portion of the research concerning the supply chain of older adults care services focuses on pricing strategies, service quality, and the coordination and optimization of these services. In the analysis of interactions between governmental entities and the supply chain for older adults care services, the evolutionary game theory approach is predominantly utilized. This analysis primarily centers on the interactions among the government and service platforms, the government and various institutions, enterprises and the government, as well as institutions and platforms. Secondly, the majority of researchers employ the Hotelling model to assess the influence of different costs on dual-channel supply chains. However, there is a notable absence of studies that apply the Hotelling model to formulate differentiated government subsidy strategies and evaluate their effects on the dual-channel supply chain for smart older adults care services. Finally, although many scholars have analyzed the multi-party cooperation model for supply chain members, in the field of smart elderly care services, most of them are comparative analysis of the optimal strategies between the decentralized mode and the centralized mode under different coordination contracts. No scholar has considered the government subsidy to compare and analyze the three cooperation mechanisms between online and offline dual channels and logistics providers.

**Table 1:** Author(s) contributions table based on literature.

Author	Supply chain	Dual channel	Smart older adults care	Gover-nment subsidy	Hotelling model	Stack-elberg game	Choice of coopera- tion strategy
Zhao et al. [10]	Y	Y	Y	N	Y	Y	N
Zhao [13]	Y	Y	Y	N	N	Y	N
Mo et al. [17]	N	N	Y	Y	N	Y	N
Chen [24]	Y	N	N	N	Y	Y	N
Ma et al. [28]	Y	N	Y	N	N	Y	N
Li et al. [29]	Y	N	N	N	N	Y	Y
Gu et al. [30]	Y	Y	N	N	Y	Y	Y
This paper	Y	Y	Y	Y	Y	Y	Y

This study investigates the dual-channel supply chain for smart care services aimed at older adults, addressing both current and future requirements while implementing a differentiated government subsidy strategy. Through case analysis, the research seeks to determine the most effective cooperation strategy. The inquiry is structured around three primary research questions: (1) the establishment of differentiated government subsidies tailored to the personalized care needs of older adults; (2) the application of Hotelling and Stackelberg game theory to assess the coordination effects of these differentiated subsidies on the dual-channel supply chain for smart older adult care services, thereby simplifying the model and aiding in the derivation of equilibrium solutions; and (3) a comparative analysis of three distinct cooperation strategies within the context of government subsidies.

The principal contributions of this paper include: (1) the design of a differentiated government subsidy mechanism that aligns with the dual-channel service offerings of the smart older adult care service supply chain; (2) an examination of the sensitivity of these differentiated subsidies to various parameters, including the pricing of older adult care services and the degree of integration between products and services; and (3) a comparative analysis of the three cooperation models under the government subsidy framework, culminating in the identification of the optimal cooperation strategy.

## 3 Problem description and symbol description

### 3.1 Problem description

**Fig 1:**
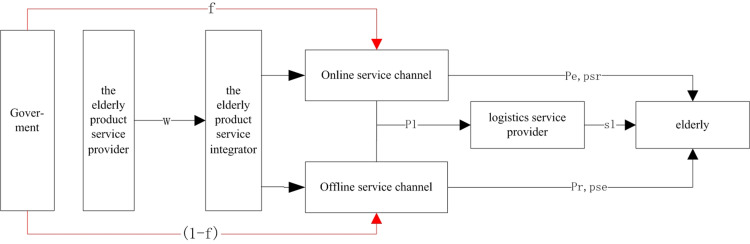
Dual channel supply chain system of older adults products and services under the government compensation mechanism.

Based on the findings of Chen et al. [31] and Zhu et al. [32], differentiated government subsidies are more effective when tailored to various methods of care for older adults, taking into account the supply and demand dynamics of the market. To encourage service integrators to enhance service levels through both online and offline channels for older adults at different stages with distinct needs, the government should provide financial incentives that are proportional to the service efforts made through these channels. This approach allows each channel to capitalize on its unique service strengths. The resulting dual-channel smart older adults care supply chain model for older adults, supported by government subsidies, is illustrated in Fig 1.

As illustrated in Fig 1, the supply chain for services aimed at older adults functions in the following manner: service providers deliver a range of comprehensive services to integrators, who subsequently disseminate these services to end-users via both online and offline platforms. The collaborative dynamics within this supply chain can be classified into three distinct modes: the first mode is characterized by a non-cooperative framework, wherein supply chain participants act autonomously; the second mode involves bilateral cooperation between online service channels and logistics providers; and the third mode encompasses a tripartite collaboration that integrates both online and offline channels alongside logistics suppliers.

This section emphasizes the importance of addressing individualized pension needs and attaining supply chain synchronization by implementing a government-subsidized dual-channel model for intelligent care services for the older adults. The following research hypotheses are developed in accordance with the research questions posed:

(1) In accordance with the fundamental tenets of the Hotelling model, it is posited that users of older adult care services are evenly distributed across the continuum of the older adult care market, represented by the interval [0,1] [23,26,30].
(2) Furthermore, to satisfy the strategic prerequisites for differentiated services, it is suggested that a competitive interaction exists between online and offline service channels, characterized by symmetric information between these modalities.
(3) Let w denote the price of the products and services provided by the older adult care service provider to the integrator. Should an older adult user opt for the offline service channel, they will incur a transportation cost denoted as t. Conversely, if they select the online service channel, they will face a search cost represented by c, in addition to a logistics service cost $c_{l}$. For the findings of this study to hold significance, it is assumed that all parameters related to costs and prices are strictly greater than zero.$\;\;\;\;\;\;\;c>0,t>0,w>0,p_{r}>0,\;\;\;\;\;\;\;p_{e}>0,p_{l}>0$ [28,30].
(4) Consistent with social emotional selection theory, the resources allocated to information services aimed at fulfilling future-oriented requirements are referred to as NFo, while the resources dedicated to emotional services that address present-oriented needs are designated as NPo [10].
(5) Considering the variations in dual-channel services and drawing upon the findings of Tsay (1999) [33], this paper asserts that the expenses associated with improving services for offline channels, online channels, and logistics are $\frac{1}{2}p{\text{s}}_{\text{r}}^{2}$, $\frac{1}{2}p{\text{s}}_{\text{e}}^{2}\;\;\;\;\;\;\;$and $\frac{1}{2}{\text{s}}_{\text{l}}^{2}$ respectively.
(6) The government provides financial incentives to offline service channels at a rate of f, which is contingent upon the fulfillment of the emotional needs of older adult users. Conversely, online service channels receive incentives at a rate of (1-f), which are predicated on the satisfaction of their informational needs [31,32]. Furthermore, the subsidy is calibrated according to the informational service efforts of the online channels and the emotional service efforts of the offline channels, with the overall subsidy intensity established at a total of 1 [34,35].

The aim of this section is to utilize a Hotelling model that incorporates both online search costs and offline transportation costs to formulate a utility function. Within the framework of a Stackelberg game pertaining to the dual-channel service supply chain for older adults, the online service channels are designated as the Leader, while the offline service channels are classified as the Follower. This analytical framework will facilitate the examination of optimal pricing strategies and the ideal degree of product and service integration across the three modes of cooperation, ultimately leading to the identification of the optimal cooperative strategy.

### 3.2 Description of the symbol

Table 2 shows the parameters related to modeling in this section.

**Table 2:** Model variables.

	sign	definition
**Decisionvariable**	$p_{r}$	Market prices for services offered through offline older adults care channels
	$p_{e}$	Market prices for services offered through online older adults care channels
	$p_{l}$	Market price for logistics services provided
	$ps_{r}$	Integration level of products and services in offline service channels
	$ps_{e}$	Integration level of products and services in online service channels
	$s_{l}$	Level of online logistics services provided
**variable**	w	Base price for products and services offered by older adults care service providers
	f	Government subsidy ratio for online service channels (where 0 < f < 1)
	$c_{l}$	Cost of logistics services for online orders
	θ	Coefficient reflecting the sensitivity of offline service channel integration level to product and service integration
	ϕ	Coefficient reflecting the sensitivity of online service channel integration level to product and service integration
	δ	Coefficient reflecting the sensitivity of online logistics service level to service integration
	$N_{Po}$	Service effort level by older adults care service providers to address current-oriented older adults needs
	$N_{Fo}$	Service effort level by older adults care service providers to address future-oriented older adults needs
	μ	Effort coefficient for older adults care service providers in meeting current orientation needs of the older adults (where 0 < μ < 1)
	t	Transportation expenses
	c	Search expenses
	x	Indifference point
	Qr	Demand for offline channel services
	Qe	Demand for online channel services
	${\text{Л}}_{\text{r}}$	Profits from offline channels
	${\text{Л}}_{\text{e}}$	Profits from online channels
	${\text{Л}}_{\text{l}}$	Profits of logistics service providers
	${\text{Л}}_{\text{el}}$	Profits from the cooperative decision-making model between online service channels and logistics service providers
	Л	Combined profits from the dual-channel supply chain

## 4 Model building

### 4.1 Utility and profit functions

Utilizing Hotelling model [23], this study develops a utility function for the dual-channel supply chain dedicated to the care of intelligent older adults, which includes both offline and online service channels supported by government subsidies. The utility functions corresponding to the online and offline service channels are delineated in Formula (1) and Formula (2), respectively.


$$U-p_{r}+\;\;\;\beta \;\;\;p_{e}-tx+f\mu N_{Po}+\theta ps_{r}$$ (1)

$$U-p_{e}+\beta p_{r}-c\left(1-x\right)+\left(1-f\right)\left(1-\mu \right)N_{Fo}+\varphi ps_{e}+\delta s_{l}$$ (2)

The demand functions for the dual-channel system are given by Formula (3) and Formula (4) respectively.


$$Q_{r}=\frac{c+fN_{Po}\mu +2p_{e}-2p_{r}-\varphi ps_{e}-\delta s_{l}+\theta ps_{r}-\left(1-f\right)\left(1-\mu \right)N_{Fo}}{c+t}$$ (3)

$$Q_{e}=\frac{t-fN_{Po}\mu -2p_{e}+2p_{r}+\varphi ps_{e}+\delta s_{l}-\theta ps_{r}+\left(1-f\right)\left(1-\mu \right)N_{Fo}}{c+t}$$ (4)

The profit functions for the dual channels and the logistics service provider are depicted in Formulas (5), (6) and (7) respectively.


$${\text{Л}}_{\text{r}}=\left({\text{p}}_{\text{r}}-\text{w}\right){\text{Q}}_{\text{r}}-\frac{1}{2}p{\text{s}}_{\text{r}}^{2}$$ (5)

$${\text{Л}}_{e}=\left(p_{e}-w-{\text{p}}_{\text{l}}\right)Q_{e}-\frac{1}{2}p{\text{s}}_{\text{e}}^{2}$$ (6)

$${\text{Л}}_{l}=\left({\text{p}}_{\text{l}}-{\text{c}}_{\text{l}}\right){\text{Q}}_{\text{e}}-\frac{1}{2}{\text{s}}_{\text{l}}^{2}$$ (7)

### 4.2 Non-cooperation service model

In the context of the Stackelberg game pertaining to the dual-channel supply chain for services aimed at older adults, the offline service channel, online service channel, and logistics service provider collaboratively establish the optimal market price for offline services, the level of product and service integration, as well as the logistics service level. The first and second partial derivatives are computed as detailed in Formulas (8)-(12) found in the Appendix. The optimal solution is represented with an asterisk “*”, while the complete decentralized decision model is denoted with a superscript “d”.

The analysis of the equations indicates that the profit function ${\text{Л}}_{r}$ associated with the offline service channel exhibits a concave relationship with respect to the variables ${\text{p}}_{\text{r}}$ and $ps_{r}$ in question. Consequently, Theorem 1 posits that, in the presence of government subsidies, there exist optimal values $p_{r}^{d\text{*}}$ and $ps_{r}^{d\text{*}}$ for these variables that maximize the profit function ${\text{Л}}_{r}$ within the context of a non-cooperative service mode.

Commence by establishing the optimal pricing strategy for offline service channels, the degree of integration between products and services, and the logistics service level. Following this, integrate these findings into Formulas (6) and (7) to obtain the $\frac{\partial^{2}{\text{Л}}_{e}{\left(p_{r}^{*}{,ps}_{r}^{*}{,s}_{l}^{*}\right)}^{d}}{\partial p_{e}}\frac{\partial^{2}{\text{Л}}_{e}{\left(p_{r}^{*}{,ps}_{r}^{*}{,s}_{l}^{*}\right)}^{d}}{\partial ps_{e}}\frac{\partial^{2}{\text{Л}}_{l}{\left(p_{r}^{*}{,ps}_{r}^{*}{,s}_{l}^{*}\right)}^{d}}{\partial p_{l}}$, $p_{e}^{d\text{*}}$, $p_{e}^{d\text{*}},\;p_{l}^{d\text{*}}$, $p_{r}^{d\text{*}}$, $ps_{r}^{d\text{*}}$ and $s_{l}^{d\text{*}}$ are shown in Formulas (13)-(18). Using Formulas (17) and (18) as a reference, the derivation process for additional formulas is presented in the Appendix.


$${\text{ps}}_{\text{e}}^{\text{d*}}=\frac{\;\;\;\varphi \;\;\;{\;\;\;\delta \;\;\;}^{2}\left(2\text{c}+2\text{t}-{\;\;\;\theta \;\;\;}^{2}\right)\left(\left(1-\;\;\;\mu \;\;\;\right)\left(1-\text{f}\right){\text{N}}_{\text{Fo}}-2\text{f }\;\;\mu \;\;\;{\text{N}}_{\text{Po}}+2\text{c}+4\text{t}-4\text{cl}\right)}{32{\left(\text{c}+\text{t}\right)}^{3}+8{\left(\text{c}+\text{t}\right)}^{2}\left(2{\;\;\;\delta \;\;\;}^{2}-{\;\;\;\theta \;\;\;}^{2}\right)-4{\;\;\;\delta \;\;\;}^{2}\left(\text{c}+\text{t}\right)\left(5{\;\;\;\theta \;\;\;}^{2}+{\;\;\;\varphi \;\;\;}^{2}\right)+2{\;\;\;\delta \;\;\;}^{2}{\;\;\;\theta \;\;\;}^{2}\left(2{\;\;\;\theta \;\;\;}^{2}+{\;\;\;\varphi \;\;\;}^{2}\right)}$$ (17)

$${\text{s}}_{\text{l}}^{\text{d*}}=\frac{\;\;\;\delta \;\;\;\left(\text{c}+\text{t}\right)\left(4\text{c}+4\text{t}-{\;\;\;\theta \;\;\;}^{2}\right)\left(\left(1-\;\;\;\mu \;\;\;\right)\left(1-\text{f}\right){\text{N}}_{\text{Fo}}-2\text{f }\;\;\mu \;\;\;{\text{N}}_{\text{Po}}+2\text{c}+4\text{t}-4\text{cl}+{\;\;\;\theta \;\;\;}^{2}\right)}{16{\left(\text{c}+\text{t}\right)}^{3}+4{\left(\text{c}+\text{t}\right)}^{2}\left(2{\;\;\;\delta \;\;\;}^{2}-{\;\;\;\theta \;\;\;}^{2}\right)-2{\;\;\;\delta \;\;\;}^{2}\left(\text{c}+\text{t}\right)\left(5{\;\;\;\theta \;\;\;}^{2}+{\;\;\;\varphi \;\;\;}^{2}\right)+{\;\;\;\delta \;\;\;}^{2}{\;\;\;\theta \;\;\;}^{2}\left(2{\;\;\;\theta \;\;\;}^{2}+{\;\;\;\varphi \;\;\;}^{2}\right)}$$ (18)

Based on the second partial derivative:

$\frac{\partial^{2}{\text{Л}}_{\text{e}}{\left({\text{p}}_{\text{r}}^{\text{*}}{\text{,ps}}_{\text{r}}^{\text{*}}{\text{,s}}_{\text{l}}^{\text{*}}\right)}^{\text{d}}}{\partial {{\text{p}}_{\text{e}}}^{2}}=-\frac{8}{4\left(\text{c}+\text{t}\right)-{\;\;\;\theta \;\;\;}^{2}}<0$ and $\frac{\partial^{2}{\text{Л}}_{\text{e}}{\left({\text{p}}_{\text{r}}^{\text{*}}{\text{,ps}}_{\text{r}}^{\text{*}}{\text{,s}}_{\text{l}}^{\text{*}}\right)}^{\text{d}}}{\partial \text{p}{{\text{s}}_{\text{e}}}^{2}}=-1$, to derive Theorem 2: In the context of government subsidies, there exist optimal values $p_{e}^{d\text{*}}$ and $p{\text{s}}_{\text{e}}^{\text{d*}}$ that maximize the profits ${\text{Л}}_{\text{e}}^{\text{d*}}$ of online service channels operating in the non-cooperative service mode.

### 4.3 Bilateral cooperation service model

This section discusses the collaborative decision-making processes between online channels and logistics service providers. The government subsidy mechanism is consistent with that of the non-cooperative framework; however, the revenue-sharing coefficient for the online service channel is represented as  λ , while that for the offline service channel is expressed as (1- λ ).

The profit functions for the dual-channel system are articulated in Formulas (19)-(20), with the results of bilateral cooperation denoted by the superscript “c”.


$${\text{Л}}_{el}=\lambda {\text{p}}_{\text{e}}{\text{Q}}_{\text{e}}-\left(\text{w}+{\text{c}}_{\text{l}}\right){\text{Q}}_{\text{e}}-\frac{1}{2}p{\text{s}}_{\text{e}}^{2}-\frac{1}{2}{\text{s}}_{\text{l}}^{2}$$ (19)

$${\text{Л}}_{\text{r}}=\left({\text{p}}_{\text{r}}-\text{w}\right){\text{Q}}_{\text{r}}+\left(1-\lambda \right){\text{p}}_{\text{e}}{\text{Q}}_{\text{e}}-\frac{1}{2}p{\text{s}}_{\text{r}}^{2}$$ (20)

As delineated in Formulas (19) and (20), the values of the variables are ascertained in accordance with Formulas (21) through (24) found in the Appendix.

An analysis of Formulas (21)-(24) reveals that: $\;\;\;\;\;\;\;\frac{\partial^{2}{\text{Л}}_{\text{r}}}{\partial {{\text{p}}_{\text{r}}}^{2}}<0$ and $\frac{\partial^{2}{\text{Л}}_{\text{r}}}{\partial \text{p}{{\text{s}}_{\text{r}}}^{2}}<0$. The profit function ${\text{Л}}_{\text{r}}^{\text{c}}$ retains its concavity with respect to the ${\text{p}}_{\text{r}}$ and $\text{p}{\text{s}}_{\text{r}}$, even in the context of bilateral cooperation. Consequently, Theorem 3 posits that the introduction of government subsidies yields optimal values ${\text{p}}_{\text{r}}^{\text{d*}}$ and ${\text{ps}}_{\text{r}}^{\text{d*}}$ that maximize ${\text{Л}}_{\text{r}}^{\text{c*}}$ within the framework of bilateral cooperation.

Employing a comparable methodology, the first and second partial derivatives of ${\text{p}}_{\text{e}}^{\text{d}}$ and ${\text{ps}}_{\text{e}}^{\text{d}}$ are computed, leading to the derivation of specific conclusions that $\frac{\partial^{2}{\text{Л}}_{el}^{c}}{\partial {p_{e}}^{2}}=-\frac{4\lambda }{\left(c+t\right)}<0$, $\frac{\partial^{2}{\text{Л}}_{el}^{c}}{\partial p{s_{e}}^{2}}=-1<0$. This analysis culminates in the formulation of Theorem 4, which asserts that in the bilateral cooperation framework augmented by government subsidies, optimal values ${\text{p}}_{\text{e}}^{\text{d*}}{\text{and ps}}_{\text{e}}^{\text{d*}}$ exist that maximize the profit ${\text{Л}}_{\text{el}}^{\text{d*}}$ associated with the online service channel.

In conclusion, the ideal pricing strategies and the levels of integration for products and services within the framework of bilateral cooperation are determined as outlined in Formulas (25)-(29).


$${\text{p}}_{\text{r}}^{\text{c*}}=\frac{2{\;\;\;\lambda \;\;\;}^{2}\left(c+t\right)\left(\text{f }\;\;\mu \;\;\;{\text{N}}_{\text{Po}}-\left(1-\text{f}\right)\left(1-\;\;\;\mu \;\;\;\right){\text{N}}_{\text{Fo}}\right)+\;\;\;\lambda \;\;\;\left(\text{c}+\text{t}\right)\left[\;\;\;\lambda \;\;\;\left({\;\;\;\varphi \;\;\;}^{2}-{\;\;\;\delta \;\;\;}^{2}\right)-\left(1-\;\;\;\lambda \;\;\;\right){\;\;\;\theta \;\;\;}^{2}-2\;\;\;\lambda \;\;\text{ t}+4\text{c}+4\text{t}+4\text{w}-4\text{cl}\right]+\text{2 }\;\;\lambda \;\;\text{ cl}\left(1-\;\;\;\lambda \;\;\;\right)\left({\;\;\;\varphi \;\;\;}^{2}-{\;\;\;\delta \;\;\;}^{2}\right)+\left(cl+w\right)\left(8c+8t-2{\;\;\;\theta \;\;\;}^{2}\right)}{2\;\;\;\lambda \;\;\;\left[\;\;\;\lambda \;\;\;\left({\;\;\;\varphi \;\;\;}^{2}-{\;\;\;\delta \;\;\;}^{2}\right)+2\left(\text{c}+\text{t}\right)\left(\;\;\;\lambda \;\;\;+2\right)-{\;\;\;\theta \;\;\;}^{2}\right]}$$ (25)

$${\text{p}}_{\text{e}}^{\text{c*}}=\frac{2\left(\text{cl}+\text{w}\right)\left(4\text{c}+4\text{t}+{\;\;\;\varphi \;\;\;}^{2}-{\;\;\;\delta \;\;\;}^{2}-{\;\;\;\theta \;\;\;}^{2}\right)+\;\;\;\lambda \;\;\;\left(\left(\text{c}+\text{t}\right)\left(4\text{w}-{\;\;\;\theta \;\;\;}^{2}\right)+2\text{c}\left(\;\;\;\lambda \;\;\;+3\text{t}\right)-4{\text{t}}^{2}\right)+2\;\;\;\lambda \;\;\;\left(c+t\right)\left(\left(1-\text{f}\right)\left(1-\;\;\;\mu \;\;\;\right){\text{N}}_{\text{Fo}}-\text{f }\;\;\mu \;\;\;{\text{N}}_{\text{Po}}\right)}{2\;\;\;\lambda \;\;\;\left[\;\;\;\lambda \;\;\;\left({\;\;\;\varphi \;\;\;}^{2}-{\;\;\;\delta \;\;\;}^{2}\right)+2\left(\text{c}+\text{t}\right)\left(\;\;\;\lambda \;\;\;+2\right)-{\;\;\;\theta \;\;\;}^{2}\right]}$$ (26)

$${\text{ps}}_{\text{r}}^{\text{c*}}=\frac{\;\;\;\theta \;\;\;\left[\left(2\text{f }\;\;\mu \;\;\;{\text{N}}_{\text{Po}}-2\left(1-\text{f}\right)\left(1-\;\;\;\mu \;\;\;\right){\text{N}}_{\text{Fo}}+2\;\;\;\lambda \;\;\;\left(\text{c}+\text{t}\right)\right)+\;\;\;\lambda \;\;\;\left({\;\;\;\varphi \;\;\;}^{2}-{\;\;\;\delta \;\;\;}^{2}\right)+2\text{c}+4\text{cl}\right]}{2\left[\;\;\;\lambda \;\;\;\left({\;\;\;\varphi \;\;\;}^{2}-{\;\;\;\delta \;\;\;}^{2}\right)+2\left(\text{c}+\text{t}\right)\left(\;\;\;\lambda \;\;\;+2\right)-{\;\;\;\theta \;\;\;}^{2}\right]}$$ (27)

$${\text{ps}}_{\text{e}}^{\text{c*}}=\frac{\;\;\;\lambda \;\;\;\;\;\varphi \;\;\;\left[\left(2\text{f }\;\;\mu \;\;\;{\text{N}}_{\text{Po}}-2\left(1-\text{f}\right)\left(1-\;\;\;\mu \;\;\;\right){\text{N}}_{\text{Fo}}-2\text{c}-4\text{t}+4\text{cl}\right)\right]}{2\;\;\;\lambda \;\;\;\left[\;\;\;\lambda \;\;\;\left({\;\;\;\varphi \;\;\;}^{2}-{\;\;\;\delta \;\;\;}^{2}\right)+2\left(\text{c}+\text{t}\right)\left(\;\;\;\lambda \;\;\;+2\right)-{\;\;\;\theta \;\;\;}^{2}\right]}$$ (28)

$${\text{s}}_{\text{l}}^{\text{c*}}=\frac{\;\;\;\lambda \;\;\;\;\;\varphi \;\;\;\left[2\text{c}-4\text{cl}-{\;\;\;\theta \;\;\;}^{2}+4\text{t}+\left(1-\text{f}\right)\left(1-\;\;\;\mu \;\;\;\right){\text{N}}_{\text{Fo}}-\text{f }\;\;\mu \;\;\;{\text{N}}_{\text{Po}}\right]}{2\left[\;\;\;\lambda \;\;\;\left({\;\;\;\varphi \;\;\;}^{2}-{\;\;\;\delta \;\;\;}^{2}\right)+2\left(\text{c}+\text{t}\right)\left(\;\;\;\lambda \;\;\;+2\right)-{\;\;\;\theta \;\;\;}^{2}\right]}.$$ (29)

### 4.4 Trilateral cooperation service mode

In this context, the dual channels and logistics providers engage in a tripartite collaboration, with the profit functions articulated in Formulas (30) to (32). The results of the cooperative decisions within this tripartite framework are indicated by the superscript “tc.”


$${\text{Л}}_{e}^{tc}=\lambda {\text{p}}_{\text{e}}{\text{Q}}_{\text{e}}-\left(\text{w}+{\text{c}}_{\text{l}}\right){\text{Q}}_{\text{e}}-\frac{1}{2}p{\text{s}}_{\text{e}}^{2}-\frac{1}{2}{\text{s}}_{\text{l}}^{2}$$ (30)

$${\text{Л}}_{r}^{tc}=\left({\text{p}}_{\text{r}}-\text{w}-{\text{c}}_{\text{l}}\right){\text{Q}}_{\text{r}}+\left(1-\lambda \right){\text{p}}_{\text{e}}{\text{Q}}_{\text{e}}-\frac{1}{2}p{\text{s}}_{\text{r}}^{2}-\frac{1}{2}{\text{s}}_{\text{l}}^{2}$$ (31)

$${\text{Л}}^{tc}={\text{Л}}_{e}^{tc}+{\text{Л}}_{r}^{tc}$$ (32)

Within the framework of tripartite cooperation that encompasses both online and offline channels, as well as logistics providers, a simultaneous game strategy is employed. The optimal pricing for services, the integration levels of products and services, and the standards of logistics services are articulated in Formulas (33) to (37).


$${\text{p}}_{\text{r}}^{\text{tc*}}=\frac{4\left(c+t\right)\left(\text{w}+{\text{c}}_{\text{l}}\right)\left(2+\;\;\;\lambda \;\;\;\right)-\left(\text{w}+{\text{c}}_{\text{l}}\right)\left(2\;\;\;\lambda \;\;\;\left({\;\;\;\delta \;\;\;}^{2}+{\;\;\;\varphi \;\;\;}^{2}\right)+2{\;\;\;\theta \;\;\;}^{2}\right)+\left(c+t\right)\left(4\;\;\;\lambda \;\;\;\left(\text{c}+\text{t}\right)-\;\;\;\lambda \;\;\;{\;\;\;\theta \;\;\;}^{2}-{\;\;\;\lambda \;\;\;}^{2}\left({\;\;\;\delta \;\;\;}^{2}+{\;\;\;\varphi \;\;\;}^{2}-{\;\;\;\theta \;\;\;}^{2}-2t\right)-2{\;\;\;\lambda \;\;\;}^{2}{\text{N}}_{\text{Fo}}\left(1-\;\;\;\mu \;\;\;\right)\left(1-f\right)+2f{\;\;\;\lambda \;\;\;}^{2}\;\;\;\mu \;\;\;{\text{N}}_{\text{Po}}\right)}{4\left(\text{c}+\text{t}\right)\left(2+\;\;\;\lambda \;\;\;\right)-2\;\;\;\lambda \;\;\;\left({\;\;\;\delta \;\;\;}^{2}+{\;\;\;\varphi \;\;\;}^{2}\right)-2{\;\;\;\theta \;\;\;}^{2}}$$ (33)

$${\text{p}}_{\text{e}}^{\text{tc*}}=\frac{4\left(c+t\right)\left(\text{w}+{\text{c}}_{\text{l}}\right)\left(2+\;\;\;\lambda \;\;\;\right)-\left(\text{w}+{\text{c}}_{\text{l}}\right)\left(2\;\;\;\lambda \;\;\;\left({\;\;\;\delta \;\;\;}^{2}+{\;\;\;\varphi \;\;\;}^{2}\right)+2{\;\;\;\theta \;\;\;}^{2}\right)+\left(c+t\right)\left(2c+4t-\;\;\;\lambda \;\;\;{\;\;\;\theta \;\;\;}^{2}+2\;\;\;\lambda \;\;\;{\text{N}}_{\text{Fo}}\left(1-\;\;\;\mu \;\;\;\right)\left(1-f\right)+2f\;\;\;\lambda \;\;\;\;\;\mu \;\;\;{\text{N}}_{\text{Po}}\right)}{4\left(\text{c}+\text{t}\right)\left(2+\;\;\;\lambda \;\;\;\right)-2\;\;\;\lambda \;\;\;\left({\;\;\;\delta \;\;\;}^{2}+{\;\;\;\varphi \;\;\;}^{2}\right)-2{\;\;\;\theta \;\;\;}^{2}}$$ (34)

$${\text{ps}}_{\text{r}}^{\text{tc*}}=\frac{\;\;\;\theta \;\;\;\left(2\;\;\;\lambda \;\;\;\left(\text{c}+\text{t}\right)+2\text{c}-\;\;\;\lambda \;\;\;\left({\;\;\;\delta \;\;\;}^{2}+{\;\;\;\varphi \;\;\;}^{2}\right)-2{\text{N}}_{\text{Fo}}\left(1-\;\;\;\mu \;\;\;\right)\left(1-\text{f}\right)+2\text{f }\;\;\mu \;\;\;{\text{N}}_{\text{Po}}\right)}{4\left(\text{c}+\text{t}\right)\left(2+\;\;\;\lambda \;\;\;\right)-2\;\;\;\lambda \;\;\;\left({\;\;\;\delta \;\;\;}^{2}+{\;\;\;\varphi \;\;\;}^{2}\right)-2{\;\;\;\theta \;\;\;}^{2}}$$ (35)

$${\text{ps}}_{\text{e}}^{\text{tc*}}=\frac{\;\;\;\lambda \;\;\;\;\;\varphi \;\;\;\left(4\text{t}+2\text{c}-{\;\;\;\theta \;\;\;}^{2}+2{\text{N}}_{\text{Fo}}\left(1-\;\;\;\mu \;\;\;\right)\left(1-\text{f}\right)-2\text{f }\;\;\mu \;\;\;{\text{N}}_{\text{Po}}\right)}{4\left(\text{c}+\text{t}\right)\left(2+\;\;\;\lambda \;\;\;\right)-2\;\;\;\lambda \;\;\;\left({\;\;\;\delta \;\;\;}^{2}+{\;\;\;\varphi \;\;\;}^{2}\right)-2{\;\;\;\theta \;\;\;}^{2}}$$ (36)

$${\text{s}}_{\text{l}}^{\text{tc*}}=\frac{\;\;\;\lambda \;\;\;\;\;\delta \;\;\;\left(4\text{t}+2\text{c}-{\;\;\;\theta \;\;\;}^{2}-2{\text{N}}_{\text{Fo}}\left(1-\;\;\;\mu \;\;\;\right)\left(1-\text{f}\right)+2\text{f }\;\;\mu \;\;\;{\text{N}}_{\text{Po}}\right)}{4\left(\text{c}+\text{t}\right)\left(2+\;\;\;\lambda \;\;\;\right)-2\;\;\;\lambda \;\;\;\left({\;\;\;\delta \;\;\;}^{2}+{\;\;\;\varphi \;\;\;}^{2}\right)-2{\;\;\;\theta \;\;\;}^{2}}$$ (37)

Further calculations of the second partial derivatives for ${\text{p}}_{\text{r}}^{\text{tc}}$, ${\text{ps}}_{\text{r}}^{\text{tc}}$, ${\text{p}}_{\text{e}}^{\text{tc}}$, ${\text{ps}}_{\text{e}}^{\text{tc}}$ yield $\frac{\partial^{2}{\text{Л}}^{\text{tc}}}{\partial {{\text{p}}_{\text{r}}}^{2}}=-\frac{4}{\text{c}+\text{t}}<0$, $\frac{\partial^{2}{\text{Л}}^{\text{tc}}}{\partial {{\text{p}}_{\text{e}}}^{2}}=-\frac{4\;\;\;\lambda \;\;\;}{\text{c}+\text{t}}<0$, $\frac{\partial^{2}{\text{Л}}^{\text{tc}}}{\partial \text{p}{{\text{s}}_{\text{r}}}^{2}}=\frac{\partial^{2}{\text{Л}}^{\text{tc}}}{\partial \text{p}{{\text{s}}_{\text{e}}}^{2}}=\frac{\partial^{2}{\text{Л}}^{\text{tc}}}{\partial {{\text{s}}_{\text{l}}}^{2}}-1<0$. Hence, Theorem 5 is established: Under the tripartite cooperation mode with government subsidies, there exist optimal values ${\text{p}}_{\text{r}}^{\text{tc*}}{\text{, ps}}_{\text{r}}^{\text{tc*}}{\text{, p}}_{\text{e}}^{\text{tc*}}{\text{, ps}}_{\text{e}}^{\text{tc*}}{\text{and s}}_{\text{l}}^{\text{tc*}}$ that maximize the total profit ${\text{Л}}^{\text{tc*}}$ of the dual-channel older adults care supply chain.

## 5 Numerical examples and simulation analysis

This section conducts a literature review concerning government subsidies, pricing strategies, and the levels of integration of products and services. It employs analytical examples to elucidate the impact of various parameters, including government subsidies, on optimal decision-making related to market pricing and service integration levels within the dual-channel supply chain for services aimed at older adults. Additionally, it provides a comparative analysis of three distinct modes of cooperation. The parameters are established as follows:$\text{w}=[4,10];{\text{c}}_{1}=[4,10];\text{c}=[4,10];\text{t}=[4,10];N_{po}=[0,10];N_{Fo}=[0,10];\theta =[0,1];\phi =[0,1];\delta =[0,1];\lambda =[0,1]$ [28,36].

### 5.1 Sensitivity analysis of the government subsidy coefficient

(1) The Influence of the Government Subsidy Coefficient on Pricing

**Fig 2:**
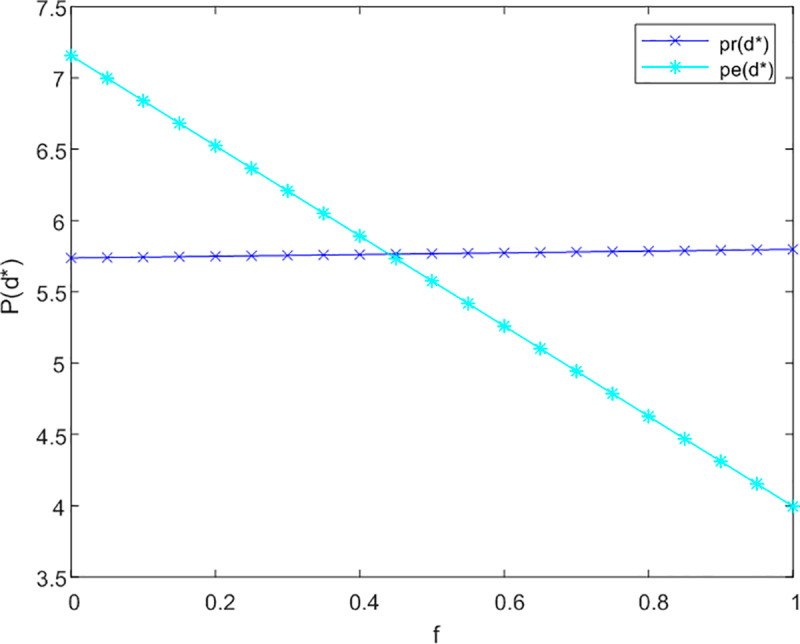
Analysis of f to P sensitivity in not cooperation.

**Fig 3:**
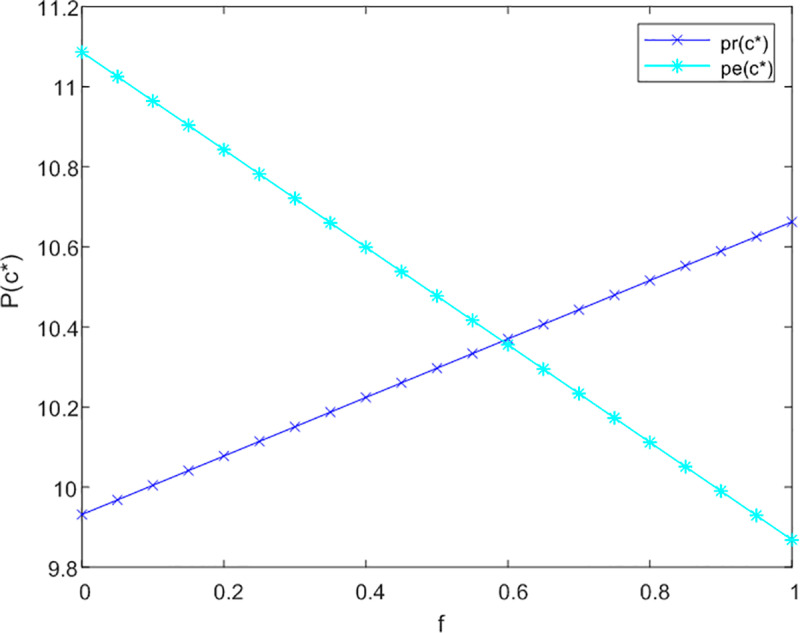
Analysis of f to P sensitivity in bilateral cooperation.

**Fig 4:**
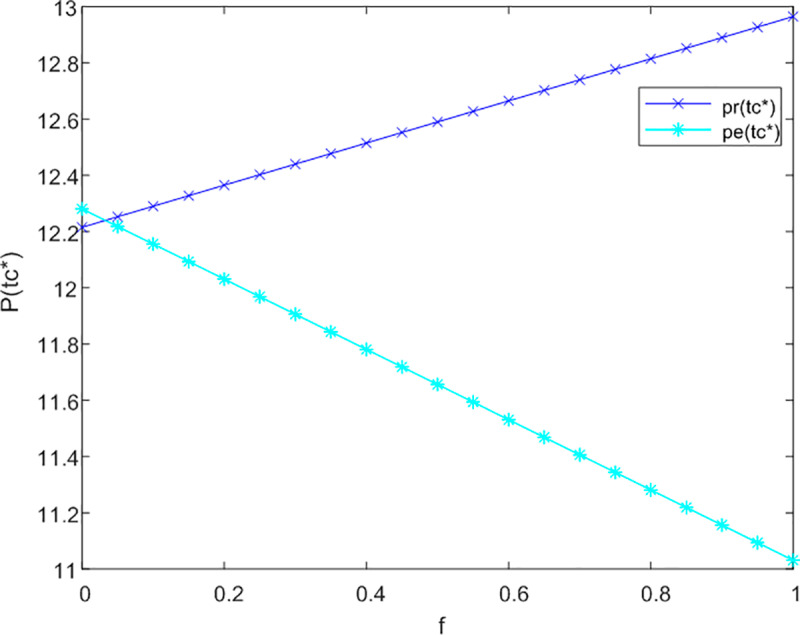
Analysis of f to P sensitivity in trilateral cooperation.

This section initially investigates the relationship between government subsidies and the pricing of both online and offline service channels, taking into account the levels of service effort dedicated to meeting the needs of current and future older adults. The sensitivity analysis illustrating the impact of government subsidies on the service prices for older adults across three cooperative models is presented in Fig 2–3 and Fig 4.

Fig 2 demonstrates that in a non-cooperative context, government subsidies are associated with an improvement in service levels for offline channels aimed at addressing the emotional needs of older adults, without influencing pricing structures. Conversely, for online channels, the introduction of subsidies results in an increase in prices. This is attributed to the fact that online channels more effectively fulfill the informational needs of older adults, leading to a rise in the subsidy coefficient (1-f) for these channels.

In Fig 3, under conditions of bilateral cooperation, the influence of the subsidy coefficient (1-f) on online channels continues to increase in a proportional manner. The interaction between online and offline channels fosters market synergies, wherein online channels offer enhanced informational services. As users’ perception of time expands, there is a growing demand for emotional companionship, prompting them to also engage with offline channels for emotional support. Following the receipt of subsidies, offline channels adjust their strategies in alignment with online channels to increase their pricing.

Fig 4 indicates that in a tripartite cooperation framework, the effects of government subsidies on both channels remain stable. Specifically, when the subsidy coefficient f surpasses 0.05 for offline channels, their optimal service price $p_{r}^{tc\text{*}}$exceeds that of online channels $p_{e}^{tc\text{*}}$. This observation underscores the necessity for government compensation mechanisms to facilitate effective tripartite service cooperation.

(2) The influence of the government subsidy coefficient on the degree of products and services

**Fig 5:**
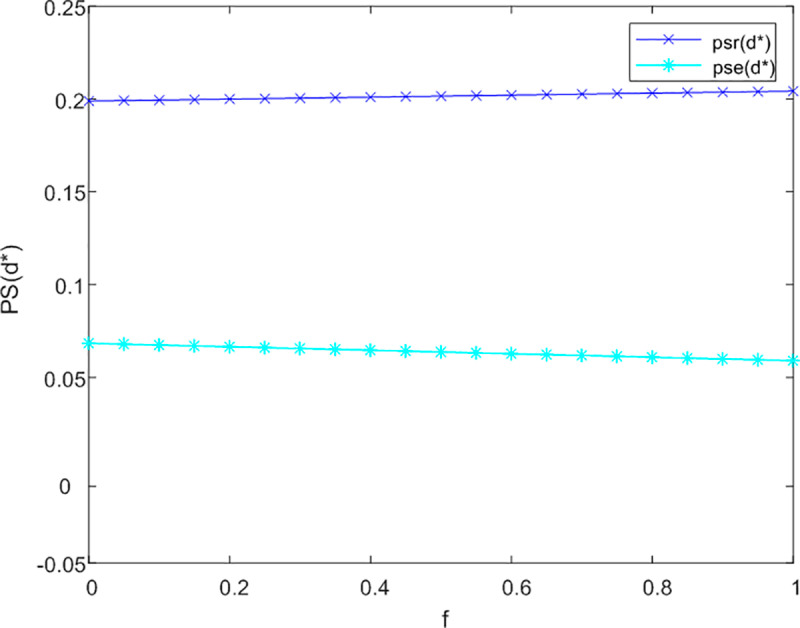
Analysis of f to PS sensitivity in not cooperation.

**Fig 6:**
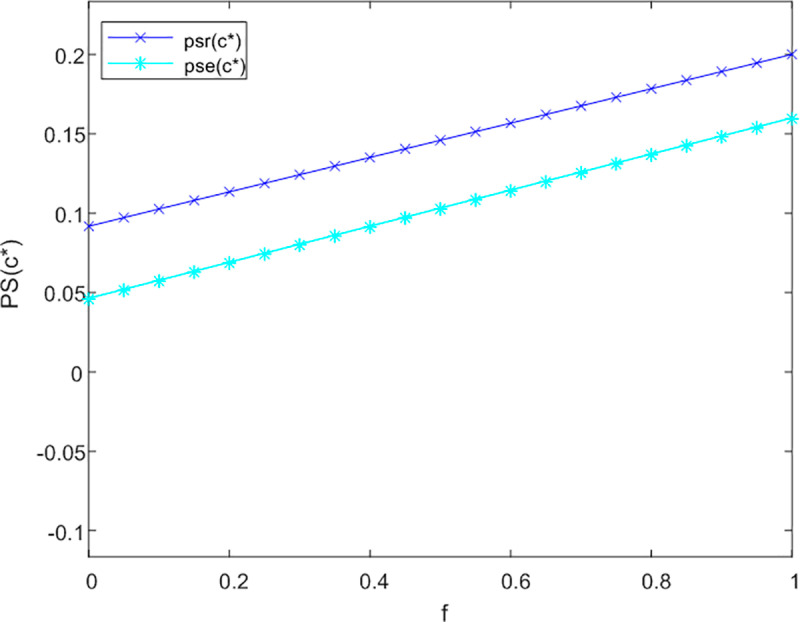
Analysis of f to PS sensitivity in bilateral cooperation.

**Fig 7:**
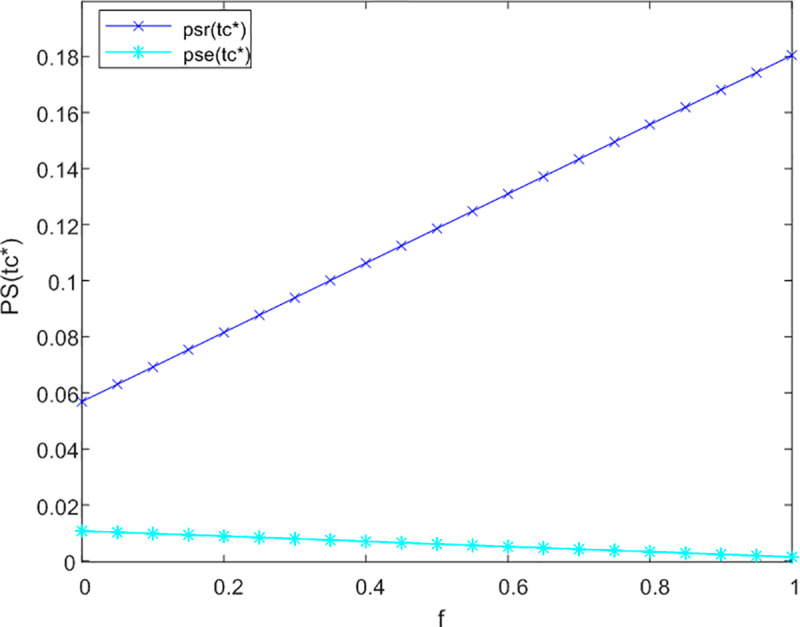
Analysis of f to PS sensitivity in trilateral cooperation.

Subsequently, the analysis examines the impact of government subsidies on the degree of integration of products and services across both online and offline service channels. The sensitivity analysis pertaining to the level of product and service integration (PS) is illustrated in Fig 5, Fig 6 and Fig 7, corresponding to non-cooperative, bilateral cooperative, and tripartite cooperative service models, respectively.

As illustrated in Figs 5 and 6, the coefficient of government subsidies has a negligible impact on the degree of integration of intelligent products and services. In a non-cooperative framework, the differences in the integration levels of online and offline service channels are nearly imperceptible. This phenomenon can be explained by the observation that, in the absence of collaboration, both online and offline service channels prioritize addressing the incremental informational and emotional needs of older adults in order to obtain additional government subsidies. Consequently, the subsidy coefficient does not exert a direct or indirect effect on the level of integration of products and services.

Under the framework of bilateral cooperation, online service channels prioritize the personalized satisfaction of care services for older adults. According to the principle of diminishing marginal utility, government subsidies may inadequately address the comprehensive development of product and service offerings within these online channels, potentially resulting in a modest decrease in PS. Conversely, offline service channels exhibit greater potential for enhancing the integration of products and services. Following the collaboration between online service channels and logistics providers, it is imperative for offline service channels to improve their product-service integration in order to attract a larger demographic of older adult users and increase their market share. Consequently, an augmentation of the government subsidy coefficient may indirectly lead to a slight enhancement in the level of product and service integration within offline service channels.

As illustrated in Fig 7, within the tripartite cooperation model, the positive impact of the government subsidy coefficient on the product-service integration level of offline service channels is significantly more pronounced than that observed in online service channels. Overall, an increase in the government subsidy coefficient correlates with an improvement in the integration level of intelligent care products and service providers for older adults.

(3) The influence of the government subsidy coefficient on profitability

**Fig 8:**
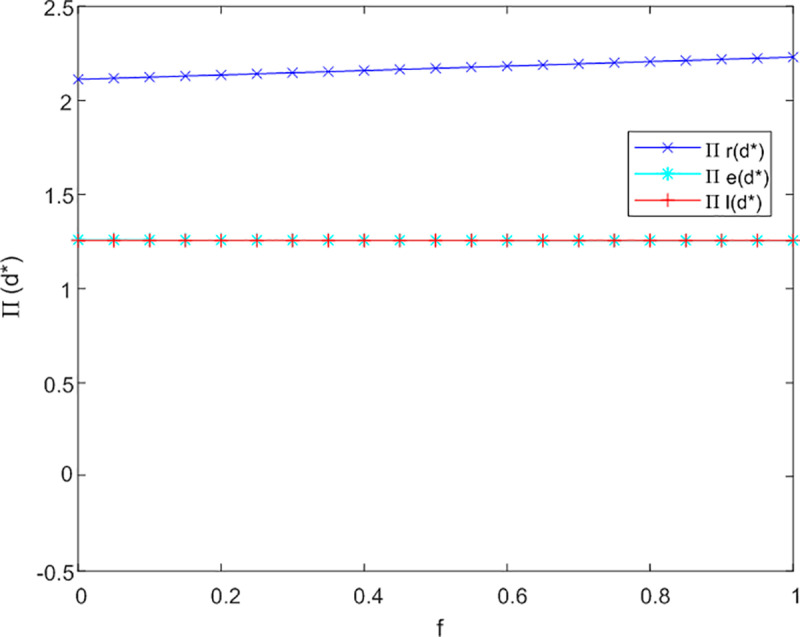
Analysis of f to Л sensitivity in not cooperation.

**Fig 9:**
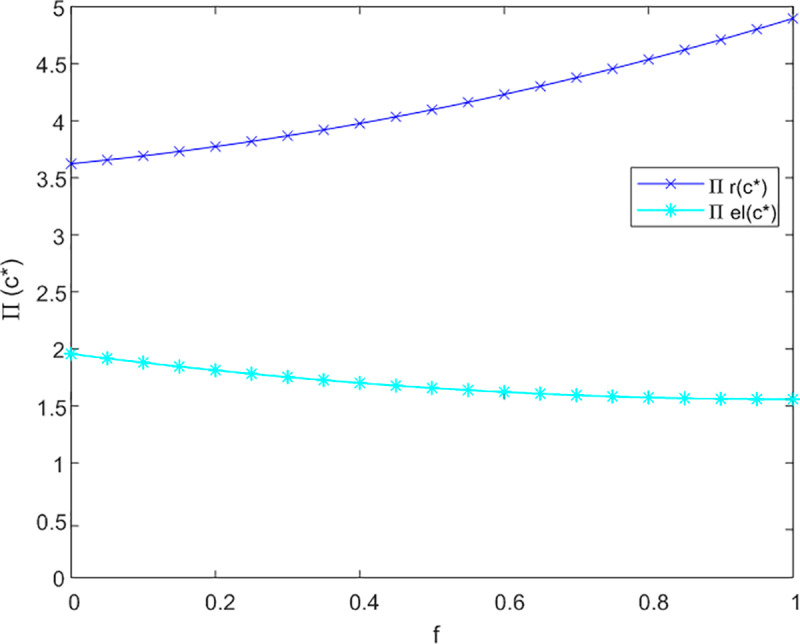
Analysis of f to Л sensitivity in bilateral cooperation.

**Fig 10:**
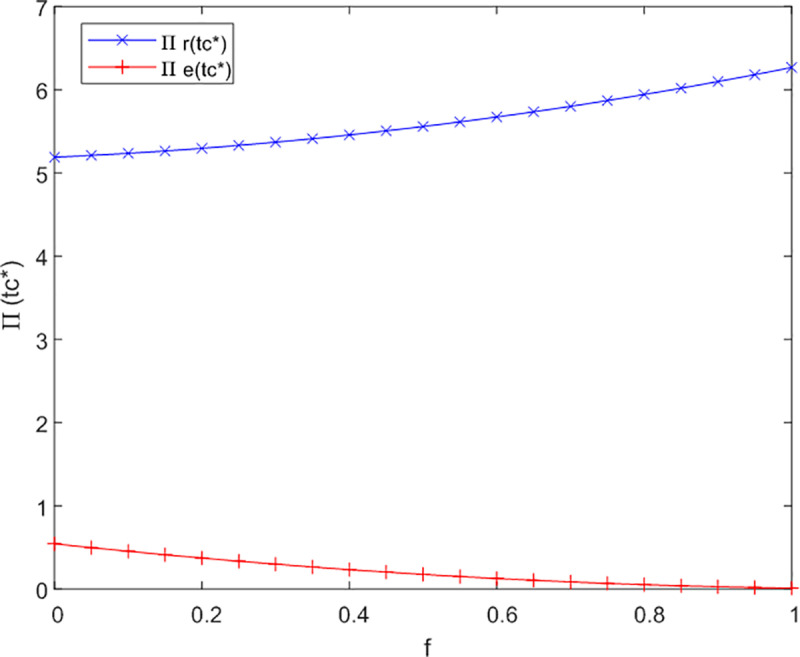
Analysis of f to Л sensitivity in trilateral cooperation.

This section of the analysis investigates the effect of the government subsidy coefficient on the profitability of the dual-channel service supply chain for older adults. The analysis of f to Л sensitivity in not cooperation, bilateral cooperation and trilateral cooperation as illustrated in Fig 8–10.

Fig 8 reveals that in a non-cooperative context, an increase in government subsidies directed towards offline service channels can enhance their profitability; however, the impact on online channels and logistics providers is comparatively minimal. This phenomenon may be attributed to the nascent stage of online care service platforms for older adults and the development of intelligent product designs, where initial investments often surpass revenues, resulting in a delay before substantial returns are realized.

As depicted in Fig 9, when bilateral cooperation is established, a rise in the government subsidy coefficient (1-f) results in a modest increase in profits for online service channels, although this increase is not as pronounced as that observed for offline channels.

Fig 10 illustrates that in a tripartite cooperative arrangement, offline service channels experience the most significant enhancement in profitability. This finding indicates that government subsidies are particularly effective in promoting quality improvements within offline service channels, thereby enabling them to broaden their service offerings, capture a larger market share, and potentially attract additional social investments at this stage of development.

### 5.2 Comparative analysis of the optimal cooperation strategy

Table 3 provides an analysis of the impact of government subsidies on optimal pricing across different service models, taking into account the effort levels (NPo and NFo) required to meet both current and future needs of care providers for older adults.

**Table 3:** Analysis of the impact of the incremental service effort level on the optimal service price under the government subsidy.

	Non-cooperation		Bilateral cooperation		Trilateral cooperation	
$N_{Fo}$	$p_{r}^{d*}$	$p_{e}^{d*}$	$p_{r}^{c*}$	$p_{e}^{c*}$	$p_{r}^{tc*}$	$p_{e}^{tc*}$
1	20.558	12.312	30.163	35.144	33.450	34.153
2	20.548	12.200	30.150	35.170	33.439	34.168
3	20.538	12.087	30.137	35.195	33.428	34.184
4	20.528	11.975	30.125	35.221	33.417	34.199
5	20.518	11.863	30.112	35.246	33.406	34.215
6	20.509	11.751	30.100	35.272	33.395	34.230
7	20.499	11.638	30.087	35.298	33.384	34.246
8	20.489	11.526	30.075	35.323	33.373	34.262
9	20.479	11.414	30.062	35.349	33.362	34.277
$N_{Po}$	$p_{r}^{d*}$	$p_{e}^{d*}$	$p_{r}^{c*}$	$p_{e}^{c*}$	$p_{r}^{tc*}$	$p_{e}^{tc*}$
1	20.593	12.716	30.208	35.052	33.489	34.097
2	20.573	12.491	30.182	35.103	33.467	34.128
3	20.554	12.267	30.157	35.154	33.445	34.159
4	20.534	12.042	30.132	35.205	33.423	34.190
5	20.514	11.817	30.107	35.257	33.402	34.221
6	20.494	11.593	30.082	35.308	33.380	34.252
7	20.474	11.368	30.057	35.359	33.358	34.283
8	20.455	11.144	30.032	35.410	33.336	34.315
9	20.435	10.919	30.007	35.462	33.314	34.346

Table 3 illustrates that the degrees of incremental information service effort and incremental emotional effort exert distinct effects on the optimal service price across different service models. In the context of offline service channels, it is evident that both the variation in the level of incremental information service effort (NFo) and the level of incremental emotional effort (NPo) result in a higher optimal service price under tripartite cooperation compared to bilateral cooperation and non-cooperative models. i.e., $p_{r}^{tc\text{*}}>p_{r}^{c\text{*}}>p_{r}^{d\text{*}}$.

From the standpoint of the evolving trend in emotional incremental effort levels, it is observed that for online service channels, the optimal strategic collaboration between these channels and logistics providers is attained through bilateral cooperation, irrespective of fluctuations in the incremental information service effort level (NFo) and the incremental emotional effort level (NPo). i.e., $p_{e}^{c\text{*}}>p_{e}^{tc\text{*}}>p_{e}^{d\text{*}}$.

Table 4 presents the findings of the analysis concerning the impact of government subsidies on the optimal degree of product and service integration across the three service models. This analysis is grounded in the effort levels (NPo and NFo) exerted by product and service providers to meet the present and future requirements of the older adults.

**Table 4:** Analysis of the impact of the incremental service effort level on the optimal product and service integration level under the government subsidy.

	Non-cooperation		Bilateral cooperation		Trilateral cooperation	
$N_{Fo}$	$ps_{r}^{d\text{*}}$	$ps_{e}^{d\text{*}}$	$ps_{r}^{c\text{*}}$	$ps_{e}^{c\text{*}}$	$ps_{r}^{tc\text{*}}$	$ps_{e}^{tc\text{*}}$
1	4.258	2.160	3.384	1.920	4.576	1.364
2	4.265	2.156	3.393	1.916	4.583	1.361
3	4.272	2.151	3.401	1.912	4.589	1.358
4	4.279	2.147	3.410	1.908	4.595	1.355
5	4.285	2.142	3.418	1.904	4.602	1.352
6	4.292	2.138	3.427	1.900	4.608	1.349
7	4.299	2.133	3.436	1.896	4.615	1.346
8	4.306	2.129	3.444	1.892	4.621	1.343
9	4.312	2.124	3.453	1.888	4.628	1.340
$N_{Po}$	$ps_{r}^{d*}$	$ps_{e}^{d*}$	$ps_{r}^{c*}$	$ps_{e}^{c*}$	$ps_{r}^{tc*}$	$ps_{e}^{tc*}$
1	4.297	2.135	3.433	1.897	4.613	1.347
2	4.293	2.137	3.428	1.900	4.609	1.348
3	4.289	2.140	3.423	1.902	4.605	1.350
4	4.285	2.143	3.418	1.904	4.602	1.352
5	4.281	2.145	3.413	1.907	4.598	1.354
6	4.277	2.148	3.408	1.909	4.594	1.355
7	4.273	2.150	3.403	1.911	4.590	1.357
8	4.269	2.153	3.398	1.914	4.587	1.359
9	4.265	2.156	3.393	1.916	4.583	1.361

Table 4 demonstrates that, within offline service channels, tripartite cooperation consistently yields superior outcomes compared to non-cooperative and bilateral cooperation models, regardless of the modifications in service providers’ approaches to addressing future-oriented demand and the degree of incremental service. Notably, when ${\text{N}}_{\text{Fo}}\epsilon \left[1,9\right]\text{, }{\text{N}}_{\text{Po}}\epsilon \left[1,9\right]$, the optimal level of product and service integration is higher in tripartite cooperation than in other forms, i.e., $\;\;\;\;\;\;\;ps_{r}^{tc\text{*}}>ps_{r}^{d\text{*}}>ps_{r}^{c\text{*}}$.

In the context of online service channels, the ideal degree of integration between products and services diminishes in the following order: non-cooperation, bilateral cooperation, and tripartite cooperation, i.e., when ${\text{N}}_{\text{Po}}\epsilon \left[1,9\right]\text{, }{\text{N}}_{\text{Po}}\epsilon \left[1,9\right],ps_{e}^{d\text{*}}>ps_{e}^{c\text{*}}>ps_{e}^{tc\text{*}}$.

**Fig 11:**
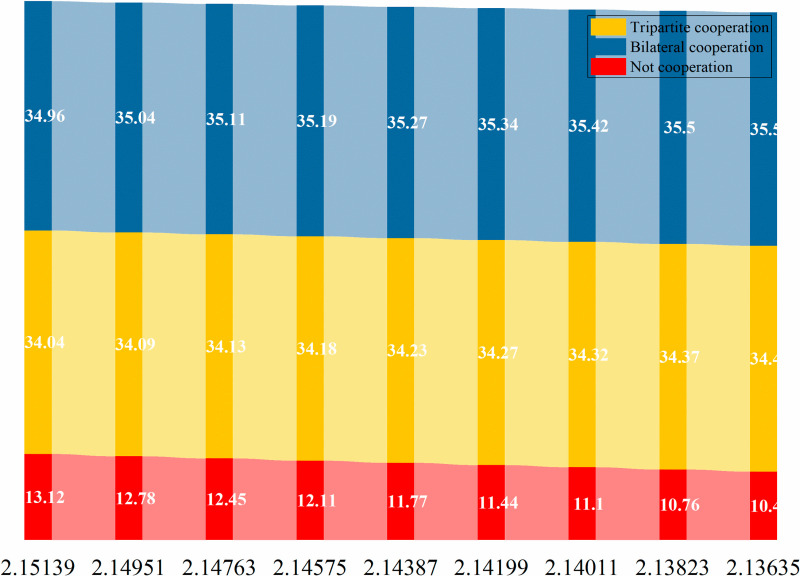
Comparative analysis of the optimal cooperation mode of offline service channels.

**Fig 12:**
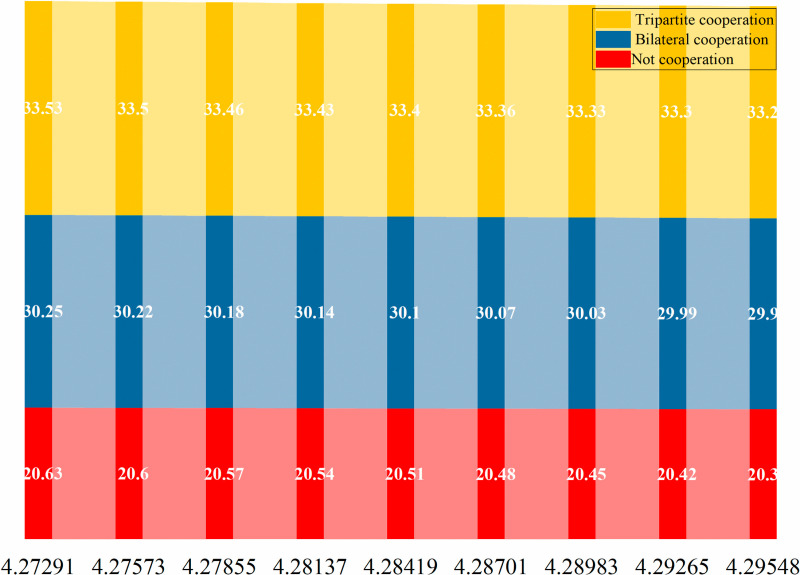
Comparative analysis of the optimal cooperation mode of online service channels.

**Fig 13:**
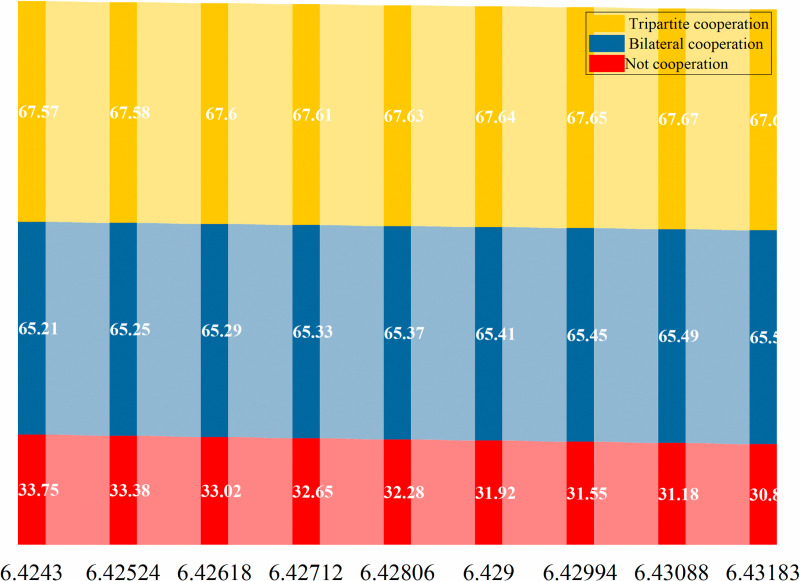
Comparative analysis of the optimal cooperation mode of dual channels.

The subsequent sections of this paper will conduct an analysis of the optimal pricing, service level, and service model in the context of varying efforts by service providers for older adults and product-service integrators to address current-oriented needs (NPo and NFo). The optimal cooperation strategies for offline service channels, online service channels, and dual channels are depicted in Fig 11–12 and Fig 13.

From Figs 11–13, it is not difficult to see that for offline service channels, the price is the best in cooperation, followed by the tripartite cooperation mode and the non-cooperation mode respectively. That is, $p_{r}^{c\text{*}}>p_{r}^{tc\text{*}}>p_{r}^{d\text{*}}$. For the online service channel, the advantages of the tripartite cooperation mode are prominent, and the service price is optimal, followed by the bilateral cooperation mode and non-cooperation mode. Namely: $p_{e}^{tc\text{*}}>p_{e}^{c\text{*}}>p_{e}^{d\text{*}}$. Finally, from the perspective of dual channel, it is consistent with the online service channel, and it still has the best performance of the tripartite cooperation mode. Namely, $p^{tc\text{*}}>p^{c\text{*}}>p^{d\text{*}}$.

## 6 Management insights

The paper utilizes the Hotelling model and the Stackelberg game model to construct a dual-channel supply chain model for smart elderly care services. Compared to previous related studies in the field of elderly care service supply chains, the introduction of the Hotelling model allows for better quantification of elderly preferences for different service channels, thereby enabling the implementation of differentiated government subsidy mechanisms for supply chain members. The development of this model provides a more comprehensive depiction of elderly needs, addressing the limitations of the Stackelberg model in analyzing elderly user behavior. Additionally, the paper designs different cost structures for online and offline channels using the model, significantly enhancing its practical applicability. Based on the research findings, the following three insights are proposed.

First, the government should adopt a proactive approach by implementing a phased and differentiated subsidy strategy that is specifically designed to address the unique characteristics of online service channels (such as platforms for smart older adults care and telemedicine institutions) and offline service channels (including older adults care facilities and community care centers). In the initial development phase, the government should prioritize the enhancement of information service functionalities for online channels, provide financial support for the operation of smart platforms, and promote research and development of smart devices. For offline channels, the government should focus on improving the quality of emotional support within community and other older adults care institutions, offer subsidies for institutional infrastructure and professional nursing personnel, and enhance the content of professional training programs. During the intermediate development phase, the government should continue to capitalize on the distinct advantages of both service types while actively fostering the integration of online and offline services. This includes supporting the connection between smart platforms and older adults care institutions to facilitate services such as telemedicine, health monitoring, and centralized care. In the final development phase, the government should conduct regular assessments of user satisfaction with online platforms and the quality of services provided by older adults care institutions, utilizing market feedback and the evolving needs of the older adults population. Furthermore, the government should dynamically adjust the intensity and distribution of subsidies to ensure the optimal allocation of resources.

Furthermore, it is imperative for the government to acknowledge its constrained capacity in enhancing the integration of products and services. Instead, it should actively involve a greater number of enterprises and social capital to bolster this integration. The government could take the initiative to create dedicated funds aimed at fostering the development of the smart older adults care sector, thereby supporting enterprises in their endeavors related to product research and development, as well as technological innovation. This support should particularly emphasize the creation of personalized and age-appropriate designs that cater to the unique needs of older adults. In parallel, the government ought to establish a health data monitoring system for the older adults, ensuring the protection of their personal information, while also conducting regular evaluations of various smart older adults care services to ensure their quality and effectiveness.

In conclusion, stakeholders within the smart older adults care service supply chain should take a proactive approach in selecting an appropriate collaboration model that aligns with the characteristics of the channels and the specific business requirements. The government can play a pivotal role by providing policy support and financial guidance to encourage supply chain participants to adopt more tripartite cooperation models, particularly in the context of smart older adults care services. By fostering partnerships among online platforms, offline services, and logistics providers, the overall quality of services and market competitiveness can be significantly enhanced.

Moreover, the government can facilitate improvements in supply chain coordination and incentive mechanisms to ensure effective communication and collaboration among all stakeholders. For instance, the establishment of a Supply Chain Relationship Management (SCRM) system could connect older adults care users with service providers and integrators, thereby creating a coordination mechanism that aligns supply with demand. This system would enable timely updates to service offerings and collaboration models based on feedback from older adults care users, ultimately enhancing the efficiency and profitability of the entire supply chain. Additionally, government subsidies have been shown to have a pronounced impact on the profitability of offline service channels, particularly within the tripartite cooperation framework. Therefore, it is advisable for the government to prioritize subsidies for offline service channels, especially those catering to financially disadvantaged older adults individuals and persons with disabilities, to ensure the provision of high-quality services.

## 7 Conclusion and study limitations

This research rigorously investigates the varying levels of incremental service efforts associated with online and offline service channels for products and services aimed at the care of older adults. Additionally, it proposes suitable levels of government subsidies to enhance these services. The study also delves into optimal cooperation strategies within the dual-channel supply chain for smart care services for the older adults. Four principal conclusions are drawn from the findings:

(1) Differentiated government subsidies significantly influence the dual-channel pricing of smart care services for older adults. An increase in the level of information incremental service effort (NFo) corresponds with a rise in the subsidy (1-f) allocated to online channels, thereby facilitating their pricing. Conversely, an increase in the level of emotional incremental service effort (NPo) leads to a higher subsidy (f) for offline channels, which similarly enhances their pricing. However, it is noted that the effect of government subsidies on the pricing of offline channels is relatively limited, with particularly weak impacts observed in non-cooperative scenarios.
(2) Government subsidies exert a negligible effect on the degree of integration between products and services. Across all three cooperation models examined, the coefficient associated with government subsidies demonstrates minimal influence on the level of product and service integration (PS).
(3) The effects of differentiated government subsidies on the profitability of the dual-channel supply chain for smart elder care services exhibit considerable variation. In non-cooperative scenarios, government subsidies result in only a modest enhancement of profits for offline service channels. Conversely, following the establishment of cooperation between the two parties, while there is a slight increase in the profitability of online service channels, the offline channels experience a more significant rise. In instances of full cooperation among all three parties, the offline service channel attains the highest level of profitability.
(4) The strengths and weaknesses of the three cooperation models are clearly delineated. The offline service channel achieves optimal pricing in scenarios involving cooperation between two parties, while the online service channel secures the best pricing outcomes when all three parties collaborate. Additionally, the omni-channel model demonstrates superior performance under tripartite cooperation. From the standpoint of achieving the optimal level of product and service integration, offline service channels consistently outperform in scenarios of tripartite cooperation, whereas online service channels exhibit optimal performance when functioning independently.

The study acknowledges its limitations, specifically its focus on the incremental demand satisfaction for older adults in relation to government subsidies, without considering other variables such as operational costs and service levels. This limitation suggests potential avenues for future research initiatives.

## Appendix

The calculate of first and second partial derivatives as shown in Formulas (8) to (12).


$$\frac{\partial {\text{Л}}_{r}^{d}}{\partial p_{r}}=\frac{c+2(p_{e}-p_{r})-\varphi ps_{e}+f\mu N_{Po}-\left(1-f\right)\left(1-\mu \right)N_{Fo}+\theta ps_{r}-s_{l}\delta -2\left(p_{r}-w\right)}{c+t}$$ (8)

$$\frac{\partial {\text{Л}}_{r}^{d}}{\partial ps_{r}}=\frac{\theta (p_{r}-w)}{c+t}-ps_{r}$$ (9)

$$\frac{\partial {\text{Л}}_{l}^{d}}{\partial s_{l}}=\frac{\delta \left(p_{l}-c_{l}\right)}{c+t}-s_{l}$$ (10)

$$\frac{\partial^{2}{\text{Л}}_{r}^{d}}{\partial {p_{r}}^{2}}=-\frac{4}{c+t}$$ (11)

$$\frac{\partial^{2}{\text{Л}}_{r}^{d}}{\partial p{s_{r}}^{2}}=-1$$ (12)

$p_{e}^{d\text{*}}$, $p_{l}^{d\text{*}}$, $p_{r}^{d\text{*}}$, $ps_{r}^{d\text{*}}$ are shown as:


$$\begin{array}{cc} & {\text{p}}_{\text{r}}^{\text{d*}}=\frac{{\;\;\;\delta \;\;\;}^{2}\left[\left(\text{c}+\text{t}\right)\left({\;\;\;\theta \;\;\;}^{2}-2\text{c}-2\text{t}\right)\left(1-\text{f}\right)\left(1-\;\;\;\mu \;\;\;\right)\right]2{\text{N}}_{\text{Fo}}-2\text{f }\;\;\mu \;\;\;{\text{N}}_{\text{Po}}\left({\left(\text{c}+\text{t}\right)}^{2}-\text{t}{\;\;\;\theta \;\;\;}^{2}\right)+{\left(\text{c}+\text{t}\right)}^{2}\left(16\text{w}-2{\;\;\;\varphi \;\;\;}^{2}+8\text{cl}-4{\;\;\;\theta \;\;\;}^{2}\right)\}}{16{\left(\text{c}+\text{t}\right)}^{3}+4{\left(\text{c}+\text{t}\right)}^{2}\left(2{\;\;\;\delta \;\;\;}^{2}-{\;\;\;\theta \;\;\;}^{2}\right)-2{\;\;\;\delta \;\;\;}^{2}\left(\text{c}+\text{t}\right)\left(5{\;\;\;\theta \;\;\;}^{2}+{\;\;\;\varphi \;\;\;}^{2}\right)+{\;\;\;\delta \;\;\;}^{2}{\;\;\;\theta \;\;\;}^{2}\left(2{\;\;\;\theta \;\;\;}^{2}+{\;\;\;\varphi \;\;\;}^{2}\right)}\\ & \text{+}\frac{{\;\;\;\delta \;\;\;}^{2}\left[\left(\text{c}+\text{t}\right)\left({\;\;\;\theta \;\;\;}^{4}+4\text{ct}+{\;\;\;\varphi \;\;\;}^{2}{\;\;\;\theta \;\;\;}^{2}-4{\;\;\;\varphi \;\;\;}^{2}\text{w}-20{\;\;\;\theta \;\;\;}^{2}\text{w}\right)+4\text{c}{\text{t}}^{2}+{\;\;\;\theta \;\;\;}^{2}\left(2{\;\;\;\varphi \;\;\;}^{2}\text{w}-4\text{ccl}-2\text{cf}+{\;\;\;\theta \;\;\;}^{2}\text{w}\right)\right]-{\;\;\;\theta \;\;\;}^{2}\text{w}{\left(\text{c}+\text{t}\right)}^{2}}{16{\left(\text{c}+\text{t}\right)}^{3}+4{\left(\text{c}+\text{t}\right)}^{2}\left(2{\;\;\;\delta \;\;\;}^{2}-{\;\;\;\theta \;\;\;}^{2}\right)-2{\;\;\;\delta \;\;\;}^{2}\left(\text{c}+\text{t}\right)\left(5{\;\;\;\theta \;\;\;}^{2}+{\;\;\;\varphi \;\;\;}^{2}\right)+{\;\;\;\delta \;\;\;}^{2}{\;\;\;\theta \;\;\;}^{2}\left(2{\;\;\;\theta \;\;\;}^{2}+{\;\;\;\varphi \;\;\;}^{2}\right)} \end{array}$$ (13)

$$\begin{array}{cc} & {\text{p}}_{\text{e}}^{\text{d*}}=\frac{{\text{N}}_{\text{Fo}}\left[8{\left(\text{c}+\text{t}\right)}^{2}\left(1-\text{f}\right)\left(1-\;\;\;\mu \;\;\;\right)\left(4\text{c}+4\text{t}+{\;\;\;\delta \;\;\;}^{2}-{\;\;\;\theta \;\;\;}^{2}\right)+2{\;\;\;\delta \;\;\;}^{2}{\;\;\;\theta \;\;\;}^{2}\left({\;\;\;\theta \;\;\;}^{2}-3\text{c}-\text{f}{\;\;\;\theta \;\;\;}^{2}-6\text{f }\;\;\mu \;\;\;\left(\text{c}+\text{t}\right)\right)\right]}{64{\left(\text{c}+\text{t}\right)}^{3}+16{\left(\text{c}+\text{t}\right)}^{2}\left(2{\;\;\;\delta \;\;\;}^{2}-{\;\;\;\theta \;\;\;}^{2}\right)-8{\;\;\;\delta \;\;\;}^{2}\left(\text{c}+\text{t}\right)\left(5{\;\;\;\theta \;\;\;}^{2}+{\;\;\;\varphi \;\;\;}^{2}\right)+4{\;\;\;\delta \;\;\;}^{2}{\;\;\;\theta \;\;\;}^{2}\left(2{\;\;\;\theta \;\;\;}^{2}+{\;\;\;\varphi \;\;\;}^{2}\right)}\\ & -\frac{\text{2f }\;\;\mu \;\;\;{\text{N}}_{\text{Fo}}{\text{N}}_{\text{Po}}[{\;\;\;\delta \;\;\;}^{2}{\;\;\;\theta \;\;\;}^{2}\left({\;\;\;\theta \;\;\;}^{2}-6c-6t\right)-16{\left(\text{c}+\text{t}\right)}^{2}\left(\text{c}+\text{t}-{\;\;\;\delta \;\;\;}^{2}\right)]}{64{\left(\text{c}+\text{t}\right)}^{3}+16{\left(\text{c}+\text{t}\right)}^{2}\left(2{\;\;\;\delta \;\;\;}^{2}-{\;\;\;\theta \;\;\;}^{2}\right)-8{\;\;\;\delta \;\;\;}^{2}\left(\text{c}+\text{t}\right)\left(5{\;\;\;\theta \;\;\;}^{2}+{\;\;\;\varphi \;\;\;}^{2}\right)+4{\;\;\;\delta \;\;\;}^{2}{\;\;\;\theta \;\;\;}^{2}\left(2{\;\;\;\theta \;\;\;}^{2}+{\;\;\;\varphi \;\;\;}^{2}\right)}\\ & +\frac{{\;\;\;\delta \;\;\;}^{2}{\;\;\;\theta \;\;\;}^{2}\left[{\;\;\;\theta \;\;\;}^{2}\left(8c+10t+4cl+8w-{\;\;\;\theta \;\;\;}^{2}\right)+4{\;\;\;\varphi \;\;\;}^{2}\left(cl+w\right)-4\left(c+t\right)\left(4cl+10w-3t\right)-20{\left(\text{c}+\text{t}\right)}^{2}\right]}{64{\left(\text{c}+\text{t}\right)}^{3}+16{\left(\text{c}+\text{t}\right)}^{2}\left(2{\;\;\;\delta \;\;\;}^{2}-{\;\;\;\theta \;\;\;}^{2}\right)-8{\;\;\;\delta \;\;\;}^{2}\left(\text{c}+\text{t}\right)\left(5{\;\;\;\theta \;\;\;}^{2}+{\;\;\;\varphi \;\;\;}^{2}\right)+4{\;\;\;\delta \;\;\;}^{2}{\;\;\;\theta \;\;\;}^{2}\left(2{\;\;\;\theta \;\;\;}^{2}+{\;\;\;\varphi \;\;\;}^{2}\right)}\\ & +\frac{16{\;\;\;\delta \;\;\;}^{2}\left[{\left(\text{c}+\text{t}\right)}^{2}\left(c+2t+w\right)-4{\;\;\;\varphi \;\;\;}^{2}\left(cl+w\right)\left(c+t\right)\right]}{64{\left(\text{c}+\text{t}\right)}^{3}+16{\left(\text{c}+\text{t}\right)}^{2}\left(2{\;\;\;\delta \;\;\;}^{2}-{\;\;\;\theta \;\;\;}^{2}\right)-8{\;\;\;\delta \;\;\;}^{2}\left(\text{c}+\text{t}\right)\left(5{\;\;\;\theta \;\;\;}^{2}+{\;\;\;\varphi \;\;\;}^{2}\right)+4{\;\;\;\delta \;\;\;}^{2}{\;\;\;\theta \;\;\;}^{2}\left(2{\;\;\;\theta \;\;\;}^{2}+{\;\;\;\varphi \;\;\;}^{2}\right)}\\ & +\frac{32{\left(\text{c}+\text{t}\right)}^{3}\left(2w+t+c\right)+32\left({\text{t}}^{3}+{\text{c}}^{3}\right)\left(t+3c\right)+4{\;\;\;\theta \;\;\;}^{2}\left({\;\;\;\theta \;\;\;}^{2}-6c-4t-4w\right){\left(\text{c}+\text{t}\right)}^{2}}{64{\left(\text{c}+\text{t}\right)}^{3}+16{\left(\text{c}+\text{t}\right)}^{2}\left(2{\;\;\;\delta \;\;\;}^{2}-{\;\;\;\theta \;\;\;}^{2}\right)-8{\;\;\;\delta \;\;\;}^{2}\left(\text{c}+\text{t}\right)\left(5{\;\;\;\theta \;\;\;}^{2}+{\;\;\;\varphi \;\;\;}^{2}\right)+4{\;\;\;\delta \;\;\;}^{2}{\;\;\;\theta \;\;\;}^{2}\left(2{\;\;\;\theta \;\;\;}^{2}+{\;\;\;\varphi \;\;\;}^{2}\right)} \end{array}$$ (14)

$$\begin{array}{cc} & {\text{p}}_{\text{l}}^{\text{d*}}=\frac{{\left(\text{c}+\text{t}\right)}^{2}[\left(\left(1-\text{f}\right)\left(1-\;\;\;\mu \;\;\;\right){\text{N}}_{\text{Fo}}-\text{f }\;\;\mu \;\;\;{\text{N}}_{\text{Po}}\right)\left(4\left(\text{c}+\text{t}\right)-{\;\;\;\theta \;\;\;}^{2}\right)+\text{cl}{\;\;\;\delta \;\;\;}^{2}[{\;\;\;\theta \;\;\;}^{2}\left(2{\;\;\;\theta \;\;\;}^{2}+{\;\;\;\varphi \;\;\;}^{2}\right)-2\left(\text{c}+\text{t}\right)\left(5{\;\;\;\theta \;\;\;}^{2}+{\;\;\;\varphi \;\;\;}^{2}\right)]}{16{\left(\text{c}+\text{t}\right)}^{3}+4{\left(\text{c}+\text{t}\right)}^{2}\left(2{\;\;\;\delta \;\;\;}^{2}-{\;\;\;\theta \;\;\;}^{2}\right)-2{\;\;\;\delta \;\;\;}^{2}\left(\text{c}+\text{t}\right)\left(5{\;\;\;\theta \;\;\;}^{2}+{\;\;\;\varphi \;\;\;}^{2}\right)+{\;\;\;\delta \;\;\;}^{2}{\;\;\;\theta \;\;\;}^{2}\left(2{\;\;\;\theta \;\;\;}^{2}+{\;\;\;\varphi \;\;\;}^{2}\right)}\\ & +\frac{{\text{t}}^{2}\left[8{\left(\text{c}+\text{t}\right)}^{2}-8cl{\;\;\;\delta \;\;\;}^{2}+\left(8{\text{c}}^{2}-2{\;\;\;\theta \;\;\;}^{2}\right)\left(2c+t\right)\right]+2{\text{c}}^{2}t\left(4c-{\;\;\;\theta \;\;\;}^{2}\right)+8{\left(\text{c}+\text{t}\right)}^{4}+{\;\;\;\theta \;\;\;}^{4}{\left(\text{c}+\text{t}\right)}^{2}-{\;\;\;\theta \;\;\;}^{2}{\left(\text{c}+\text{t}\right)}^{3}}{16{\left(\text{c}+\text{t}\right)}^{3}+4{\left(\text{c}+\text{t}\right)}^{2}\left(2{\;\;\;\delta \;\;\;}^{2}-{\;\;\;\theta \;\;\;}^{2}\right)-2{\;\;\;\delta \;\;\;}^{2}\left(\text{c}+\text{t}\right)\left(5{\;\;\;\theta \;\;\;}^{2}+{\;\;\;\varphi \;\;\;}^{2}\right)+{\;\;\;\delta \;\;\;}^{2}{\;\;\;\theta \;\;\;}^{2}\left(2{\;\;\;\theta \;\;\;}^{2}+{\;\;\;\varphi \;\;\;}^{2}\right)} \end{array}$$ (15)

$$\begin{array}{cc} & {\text{ps}}_{\text{r}}^{\text{d*}}=\frac{\;\;\;\theta \;\;\;[4{\left(\text{c}+\text{t}\right)}^{2}\left(4\left(\text{c}+\text{t}\right)-{\;\;\;\theta \;\;\;}^{2}\right)+{\;\;\;\delta \;\;\;}^{2}[\left(4\left(\text{c}+\text{t}\right)-2{\;\;\;\theta \;\;\;}^{2}\right)\left(\text{f }\;\;\mu \;\;\;{\text{N}}_{\text{Po}}-\left(1-\text{f}\right)\left(1-\;\;\;\mu \;\;\;\right){\text{N}}_{\text{Fo}}\right)]}{32{\left(\text{c}+\text{t}\right)}^{3}+8{\left(\text{c}+\text{t}\right)}^{2}\left(2{\;\;\;\delta \;\;\;}^{2}-{\;\;\;\theta \;\;\;}^{2}\right)-4{\;\;\;\delta \;\;\;}^{2}\left(\text{c}+\text{t}\right)\left(5{\;\;\;\theta \;\;\;}^{2}+{\;\;\;\varphi \;\;\;}^{2}\right)+2{\;\;\;\delta \;\;\;}^{2}{\;\;\;\theta \;\;\;}^{2}\left(2{\;\;\;\theta \;\;\;}^{2}+{\;\;\;\varphi \;\;\;}^{2}\right)}\\ & \text{+}\frac{\;\;\;\theta \;\;\;{\;\;\;\delta \;\;\;}^{2}[{\;\;\;\theta \;\;\;}^{2}\left({\;\;\;\theta \;\;\;}^{2}+{\;\;\;\varphi \;\;\;}^{2}-6\text{c}-4\text{t}-4\text{cl}\right)+2\left(4\text{cl}+{\;\;\;\varphi \;\;\;}^{2}\right)\left(\text{c}+\text{t}\right)+4\text{ct}]}{32{\left(\text{c}+\text{t}\right)}^{3}+8{\left(\text{c}+\text{t}\right)}^{2}\left(2{\;\;\;\delta \;\;\;}^{2}-{\;\;\;\theta \;\;\;}^{2}\right)-4{\;\;\;\delta \;\;\;}^{2}\left(\text{c}+\text{t}\right)\left(5{\;\;\;\theta \;\;\;}^{2}+{\;\;\;\varphi \;\;\;}^{2}\right)+2{\;\;\;\delta \;\;\;}^{2}{\;\;\;\theta \;\;\;}^{2}\left(2{\;\;\;\theta \;\;\;}^{2}+{\;\;\;\varphi \;\;\;}^{2}\right)} \end{array}$$ (16)

The values of ${\text{p}}_{\text{r}}$ and $\text{p}{\text{s}}_{\text{r}}$ are determined as presented in Formulas (21)-(24) are shown as:


$$\frac{\partial {\text{Л}}_{r}^{c}}{\partial p_{r}}=\frac{c+4p_{e}-4p_{r}+2w-\varphi ps_{e}+f\mu N_{Po}-\left(1-f\right)\left(1-\mu \right)N_{Fo}+\theta ps_{r}-s_{l}\delta -2\lambda p_{e}}{c+t}$$ (21)

$$\frac{\partial {\text{Л}}_{r}^{c}}{\partial ps_{r}}=\frac{\theta \left[\left(p_{r}-w\right)-p_{e}\left(1-\lambda \right)\right]}{c+t}-ps_{r}$$ (22)

$$\frac{{{\partial \text{Л}}^{2}}_{\text{r}}^{\text{c}}}{\partial {{\text{p}}_{\text{r}}}^{2}}=-\frac{4}{\text{c}+\text{t}}$$ (23)

$$\frac{{{\partial \text{Л}}^{2}}_{\text{r}}^{\text{c}}}{\partial \text{p}{{\text{s}}_{\text{r}}}^{2}}=-1$$ (24)

## Supporting information

S1 Data Data availability. (XLS)
